# Pain Catastrophizing Is Associated with Knee Function in Physically Active Individuals with Patellofemoral Pain: A Cross-Sectional Study

**DOI:** 10.3390/healthcare14193212

**Published:** 2026-09-29

**Authors:** Ahmed Alalawi, Roaa Sroge, Abdulaziz Awali

**Affiliations:** Department of Medical Rehabilitation Sciences, Faculty of Applied Medical Sciences, Umm Al-Qura University, Makkah 24382, Saudi Arabia; rasroge@uqu.edu.sa (R.S.); amawali@uqu.edu.sa (A.A.)

**Keywords:** patellofemoral pain, pain catastrophizing, anterior knee pain scale, psychological factors, knee function

## Abstract

**Background/Objectives:** Patellofemoral pain (PFP) is a common and often persistent knee condition traditionally understood in biomechanical terms, though psychological factors are increasingly recognized as relevant. Pain catastrophizing has attracted particular attention, but most evidence relies on univariable associations, is drawn from female-dominated samples, and rarely tests whether catastrophizing relates to knee function after adjustment for other relevant factors. This study examined the associations between pain catastrophizing, perceived stress, body mass index (BMI), and knee pain history and knee function in physically active individuals with PFP, and whether catastrophizing remained associated with function after adjustment for the other included factors. **Methods:** In this cross-sectional study, 55 physically active individuals with symptoms of PFP completed the Anterior Knee Pain Scale (AKPS), the Pain Catastrophizing Scale, and the Perceived Stress Scale. BMI and knee pain history were collected. Univariable analyses were followed by a multivariable model including all four prespecified predictors. **Results:** In univariable analyses, higher pain catastrophizing (β = −0.48, *p* < 0.001), higher perceived stress (β = −0.70, *p* = 0.004), and higher BMI (β = −0.58, *p* = 0.032) were each associated with lower AKPS scores. After adjustment, only pain catastrophizing remained significant (β = −0.36, 95% CI −0.60 to −0.11, *p* = 0.005). The final adjusted model explained 28% of the variance in AKPS scores. **Conclusions:** Pain catastrophizing remained associated with poorer knee function after adjustment for perceived stress, BMI, and knee pain history, suggesting that it may warrant consideration when assessing physically active individuals with PFP.

## 1. Introduction

Patellofemoral pain (PFP) is pain around or behind the patella that is exacerbated when it is loaded during weight bearing, particularly during squatting, stair ambulation, running, hopping or jumping [1]. The prevalence of PFP in the general population was estimated to be 22.7% annually, and 28.9% among adolescents [2]. Far from being self-limiting, PFP frequently persists. A study found that 40% of participants with PFP had not fully recovered at 12 months [3], and over half of those followed for 5–8 years continued to report significant symptoms [4]. In addition, a longer duration of symptoms has been shown repeatedly to be associated with poorer prognosis [3,5].

PFP has been mainly viewed within a biomechanical framework, with symptoms associated with patellofemoral joint loading and associated movement or muscle impairments [6]. Although this framework remains clinically relevant, it does not fully account for the variation in pain and disability observed among individuals. Increasing attention has therefore been directed toward the psychological and behavioral dimensions of PFP. A systematic review found that anxiety, depression, catastrophizing, and fear of movement may be elevated in people with PFP and may correlate with pain and reduced physical function, although the evidence was derived from a limited number of studies [7]. More recently, an international consensus identified pain catastrophizing, pain self-efficacy, and fear-avoidance beliefs as the only psychological factors considered clinically important for examination and treatment planning [8]; all three were also prioritized for further research [8].

Among these constructs, pain catastrophizing has received particular attention. Yet the evidence in PFP remains mixed and methodologically limited. Priore et al. [9] found greater catastrophizing among women with PFP than among pain-free controls, but no significant association was found with performance on objective functional tests. In a multivariable study, catastrophizing was not retained after kinesiophobia and depression were considered [10]. By contrast, Maclachlan et al. [11] retained catastrophizing alongside kinesiophobia, pain self-efficacy, and knee pressure pain threshold in a model that explained 40% of the variance in PFP-related function. Much of the available evidence has involved female-predominant or selected clinical samples. General perceived stress has received even less attention than pain-specific constructs, leaving uncertainty about whether catastrophizing is associated with knee function after other psychological and clinical factors are considered. More recently, a systematic review of prognostic factors in PFP found that psychological factors were among the least frequently studied domains, highlighting the need for further research examining their relationship with clinical outcomes [12].

Accordingly, this study aimed to examine the associations of pain catastrophizing, perceived stress, body mass index (BMI), and knee pain history with self-reported knee function in physically active individuals with symptoms of PFP. A secondary aim was to determine whether pain catastrophizing remained associated with knee function after adjustment for the other included factors. It was hypothesized that greater catastrophizing, higher perceived stress, higher BMI, and recurrent knee pain would be associated with poorer knee function, and that catastrophizing would remain associated with function after adjustment for the other included factors.

## 2. Materials and Methods

### 2.1. Study Design

This was a cross-sectional observational study investigating the associations between psychological, clinical, and physical factors and knee function in physically active individuals with PFP. Data were collected through a self-administered online survey comprising study-specific demographic, training, and PFP screening questions, together with previously validated Arabic patient-reported outcome measures. The study was reported in accordance with the Strengthening the Reporting of Observational Studies in Epidemiology (STROBE) guidelines for cross-sectional studies [13], and the completed checklist is provided as Supplementary File S1. The study was conducted in accordance with the Declaration of Helsinki and was approved by the Biomedical Research Ethics Committee (HAPO-02-K-012-2025-06-2820).

### 2.2. Participants and Recruitment

Participants were recruited using a convenience sampling approach through an online self-administered questionnaire distributed to physically active adults and gym users. The online survey comprised study-specific screening and demographic questions together with three validated patient-reported measures. The screening questions were used to identify participants with symptoms consistent with PFP and were based on the consensus definition of PFP [1,14], namely pain around or behind the patella aggravated by activities that load the patellofemoral joint during weight bearing on a flexed knee, such as squatting, stair use, running, or jumping [1,14]. The inclusion criteria were: (1) age 18–40 years; (2) participation in gym-based exercise at least two days per week; and (3) self-reported symptoms consistent with PFP. The age restriction was intended to reduce the likelihood that symptoms were attributable to age-related degenerative knee conditions. These screening questions were used for eligibility purposes and did not constitute a validated diagnostic questionnaire or a clinical diagnosis of PFP.

The exclusion criteria were as follows: (1) age outside the specified range; (2) not meeting the symptom criteria for PFP; or (3) incomplete responses for the primary outcome or key predictor variables. All participants provided informed consent prior to completing the questionnaire. A total of 62 records were screened. Seven were excluded before analysis: three because age was outside the prespecified 18–40-year range, two because they did not meet the prespecified PFP symptom criteria, one because of incomplete outcome data, and one because of incomplete data for a key predictor variable. The final analysis therefore included 55 participants. No otherwise eligible observations were excluded from the primary analysis on the basis of regression influence or model diagnostics.

The survey was administered using Google Forms (Google LLC, Mountain View, CA, USA), and the questionnaire link was distributed through social media and word of mouth among regular gym users. Data were collected between November 2025 and May 2026. Before accessing the survey questions, participants viewed an electronic information and consent statement and were required to provide consent before proceeding. Responses were stored securely and were accessible only to the research team.

### 2.3. Outcome

The primary outcome was knee function measured by the Anterior Knee Pain Scale (AKPS). The AKPS is a 13-item patient-reported outcome measure designed to evaluate subjective symptoms and functional limitations of PFP [15]. The scale consists of items about activities and symptoms including limping, support, ambulation, stair descending, squatting, running, jumping, prolonged sitting, pain, swelling, patellar symptoms, muscle atrophy, and limitation of knee flexion [16]. It provides a total score from 0 to 100 (higher scores indicate better knee function and fewer symptoms). The Arabic version of the AKPS was used in the present study and has been previously shown to be valid and reliable in Arabic-speaking individuals with PFP [16,17,18].

### 2.4. Candidate Predictors

The candidate predictors were selected a priori, as they are clinically relevant and previous evidence indicates that psychological and symptom-related factors may be associated with pain and function in people with PFP [7]. The candidate predictors consisted of pain catastrophizing, perceived stress, BMI and history of knee pain.

Pain catastrophizing was measured with the Pain Catastrophizing Scale (PCS), a 13-item self-reported measure that evaluates exaggerated negative thoughts and feelings about pain [19]. Items are rated on a 5-point scale from 0 to 4, producing a total score ranging from 0 to 52, with higher scores indicating greater pain catastrophizing. The Arabic version of the PCS was used in this study and has demonstrated validity and reliability in Arabic-speaking individuals [20].

The 10-item Perceived Stress Scale (PSS) was used to assess perceived stress, which reflects the perceived level of stress, unpredictability, lack of control and being overwhelmed by situations in one’s life [21]. Items are rated on a 5-point scale from 0 to 4, producing a total score ranging from 0 to 40, with higher scores reflecting greater perceived stress. The Arabic version of the PSS has already been found to have good psychometric properties and was used in the current study [22].

BMI was calculated from self-reported weight and height. BMI was included as an a priori anthropometric covariate because body mass may influence lower-limb loading during weight-bearing activities and anthropometric factors are commonly examined in relation to PFP and knee function [23].

History of knee pain was assessed by asking participants whether their current episode of anterior knee pain was their first or whether they had experienced previous episodes. Knee pain history was categorized as first-time or recurrent knee pain for analysis.

### 2.5. Statistical Analysis

Participant characteristics were summarized using descriptive statistics, and continuous variables were checked for normality using the Shapiro–Wilk test [24].

Linear regression analyses were used to examine associations between candidate predictors and knee function. Multivariable linear regression has been used to examine associations between clinical and psychological factors and patient-reported outcomes, consistent with previous PFP research [25]. The dependent variable was the total AKPS score, where a higher score represented better knee function and fewer symptoms.

#### 2.5.1. Univariable Models

The outcome and candidate predictors were selected prior to analysis, based on clinical relevance and previous literature regarding the factors associated with PFP [7,23,26,27]. Total AKPS score was used as the dependent variable. Four candidate predictors were examined including pain catastrophizing, perceived stress, BMI and knee pain history. The individual predictors were each examined in a separate linear regression model to provide unadjusted estimates of their associations with knee function. Knee pain history was treated as a categorical variable, with first-time knee pain serving as the reference category. Univariable model results were presented using unstandardized regression coefficients (β), standard errors, 95% confidence intervals, *p*-values, and R^2^ values.

#### 2.5.2. Multivariable Model

To evaluate whether each candidate predictor remained associated with knee function after adjustment for the other included covariates, a multivariable linear regression model including pain catastrophizing, perceived stress, BMI, and knee pain history was fitted [28]. No formal a priori sample-size calculation was conducted. The sample size was determined by feasibility and the number of eligible participants with complete data recruited during the study period. To reduce the risk of overfitting, the model was restricted to four prespecified predictors selected on clinical and theoretical grounds [29,30]. With 55 participants, this corresponded to approximately 14 observations per predictor. This ratio was used to guide model parsimony and should not be interpreted as evidence of adequate statistical power. Accordingly, the regression analyses were considered exploratory, and interpretation emphasized the magnitude and precision of the effect estimates, including 95% confidence intervals, rather than statistical significance alone. All predictors were retained in the adjusted model, and no automated variable-selection procedure was applied. Multicollinearity was assessed using the variance inflation factor (VIF) [31]. In this dataset, VIF values ranged from 1.09 to 1.20, indicating no evidence of substantial multicollinearity among the included predictors.

Regression assumptions and model diagnostics were assessed before interpretation of the multivariable model [32]. Linearity and homoscedasticity were evaluated using residual-versus-fitted and scale-location plots, with homoscedasticity additionally assessed using the Breusch–Pagan test [32]. Residual normality was assessed using a normal Q-Q plot and the Shapiro–Wilk test [24]. Potentially influential observations were examined using studentized residuals, leverage values, and Cook’s distance, with screening thresholds of 4/n for Cook’s distance and 2k/n for leverage [33], where n is the sample size and k is the number of model parameters including the intercept. Diagnostic measures were used to assess model adequacy and were not used as criteria for excluding observations. As a sensitivity analysis, the adjusted model was refitted after individually and collectively removing observations flagged by the influence diagnostics to assess whether the main association was materially dependent on these observations.

Results from the multivariable model were reported using unstandardized regression coefficients (β), standard errors, 95% confidence intervals, *p*-values, and adjusted R^2^. Statistical significance was set at *p* < 0.05. All analyses were performed in R statistical software, version 4.5.2.

## 3. Results

### 3.1. Characteristics of Participants

Of the 55 participants, 49 (89%) were male and 6 (11%) were female. The mean age of the participants was 27.38 years (SD = 7.16), and the mean BMI was 26.06 kg/m^2^ (SD = 5.17), as shown in Table 1. The mean score for pain catastrophizing and perceived stress was 12.25 (SD = 10.67) and 16.13 (SD = 5.66), respectively. The overall degree of knee function within the sample was generally moderate to good, with a mean AKPS score of 78.24 (SD = 10.41).

### 3.2. Associations Between Candidate Predictors and AKPS Outcome

#### 3.2.1. Unadjusted Associations Between Candidate Predictors and AKPS Outcome

The unadjusted associations between the candidate predictors and AKPS outcome are reported in Table 2. Higher pain catastrophizing was significantly associated with lower AKPS scores (β = −0.48, 95% CI: −0.72 to −0.25, *p* < 0.001), indicating worse knee function with greater pain catastrophizing. Similarly, higher perceived stress (β = −0.70, 95% CI: −1.17 to −0.23, *p* = 0.004) and higher BMI (β = −0.58, 95% CI: −1.11 to −0.05, *p* = 0.032) were associated with poorer knee function. Recurrent knee pain history was not significantly associated with AKPS outcome in the unadjusted analysis (*p* = 0.227).

#### 3.2.2. Adjusted Associations Between Candidate Predictors and AKPS Outcome

The adjusted multivariable regression model is shown in Table 3. Pain catastrophizing remained associated with AKPS outcome after adjustment for perceived stress, BMI, and knee pain history (β = −0.36, 95% CI: −0.60 to −0.11, *p* = 0.005). In contrast, perceived stress, BMI, and recurrent knee pain history were not associated with AKPS scores after adjustment for the other included covariates. The final adjusted model explained 28% of the variance in AKPS scores (adjusted R^2^ = 0.28).

Model diagnostics indicated no substantial violation of the assumptions of linearity, residual normality, or homoscedasticity. Residuals did not significantly deviate from normality (Shapiro–Wilk W = 0.97, *p* = 0.16), and the Breusch–Pagan test provided no evidence of heteroscedasticity (BP = 2.86, df = 4, *p* = 0.58). No observation had an absolute studentized residual greater than 3. Although three observations exceeded the conventional screening threshold for Cook’s distance (4/n = 0.07) and three exceeded the leverage threshold (2k/n = 0.18), the maximum Cook’s distance was 0.11. Sensitivity analyses excluding observations flagged by Cook’s distance or leverage produced similar estimates for pain catastrophizing (β range = −0.41 to −0.30), with the association remaining statistically significant across all sensitivity models (*p* = 0.005–0.021).

## 4. Discussion

### 4.1. Summary of Main Findings

The purpose of this study was to investigate the associations between psychological, physical and clinical measures and knee function in physically active adults presenting with PFP. The primary finding was that pain catastrophizing remained associated with knee function after adjustment for perceived stress, BMI, and knee pain history. Univariable analyses revealed that greater pain catastrophizing, greater perceived stress, and greater BMI were all associated with poorer AKPS scores. After adjustment, only pain catastrophizing was statistically significant. The final multivariable model accounted for 28% of variance in AKPS scores, while the remaining variability was not explained by the included factors. The findings indicate an association between catastrophizing and self-reported knee function but do not establish its direction.

### 4.2. Pain Catastrophizing and Knee Function

Pain catastrophizing was the only candidate predictor that remained associated with knee function after adjustment for perceived stress, BMI, and knee pain history. Each one-point increase in PCS score was associated with an estimated 0.36-point reduction in the AKPS score, indicating poorer self-reported knee function. This finding supports consideration of PFP within a broader biopsychosocial framework, in which pain-related cognitive responses may be associated with the experience of symptoms and functional limitations.

The present finding is broadly consistent with a systematic review indicating that catastrophizing may be associated with pain and reduced function in people with PFP, although the available evidence was limited and heterogeneous [7]. Maclachlan et al. [11] also identified catastrophizing as one component of a psychological and somatosensory model that explained 40% of the variance in PFP-related function. However, an earlier study found that, although catastrophizing was higher in individuals with more severe symptoms, it was not retained as an independent predictor of disability after other psychological factors were considered [10]. Kinesiophobia and depression were retained in the final model. These contrasting findings may partly reflect differences in outcome measurement and model composition. The present study assessed knee function using the AKPS, whereas Maclachlan et al. [10] used the Knee Injury and Osteoarthritis Outcome Score and included several related psychological constructs, including kinesiophobia and depression. However, because kinesiophobia, pain self-efficacy, and other psychological constructs were not included in the present model, the association observed for catastrophizing should not be interpreted as independent of the broader psychological profile.

The relationship between catastrophizing and function may depend on whether function is measured through self-report or objective performance measures. In women with PFP, Priore et al. [9] observed a higher level of catastrophizing than in pain-free controls, but catastrophizing was not associated with objective functional test performance. The AKPS used in this study, by contrast, is a self-reported measure that assesses symptoms and functional limitations associated with activities, such as squatting, stair use, running and prolonged sitting. Differences in the activities assessed may help explain the contrasting findings. Priore et al. [9] evaluated hopping, step-down and dynamic balance, whereas the AKPS captures a broader range of self-reported limitations, including stair use, running, and prolonged sitting. Similarly, Gilgallon et al. [34] found that pain catastrophizing was not associated with objectively measured steps per day or moderate-to-vigorous physical activity in individuals with PFP, whereas pain self-efficacy was associated with both measures. This further suggests that different psychological constructs may relate differently to self-reported function and objectively measured physical activity.

This distinction is clinically significant. A global consensus found that three factors were relevant to the clinical examination, treatment planning and prognosis of PFP: pain catastrophizing, pain self-efficacy, and fear-avoidance beliefs [8]. Clinical guidance also recommends assessing psychological factors that could affect prognosis and rehabilitation decisions [14]. If these are present, tailored education, reassurance, graded exposure, cognitive-behavioral approaches or referral may be considered [14]. Because the present study was cross-sectional, the temporal direction of the association cannot be determined. Greater catastrophizing could be associated with poorer perceived knee function, but poorer function may also contribute to greater catastrophizing.

### 4.3. Perceived Stress, BMI, and Knee Pain History

Perceived stress was associated with poorer knee function in the unadjusted analysis, but not after adjustment. General stress has received less attention in PFP research than pain-specific constructs such as catastrophizing and kinesiophobia. Jensen et al. [35] found greater mental distress and poorer self-perceived health among individuals with long-term PFP than among pain-free controls. Mental distress was also correlated with greater knee pain and poorer self-reported knee function. In a broader population aged 50 years or older, increasing stress was associated with progressively greater odds of chronic knee pain [36], although that population differed substantially from the present sample. The weakening of the association after adjustment may reflect overlap between general stress and pain-specific cognitions, particularly catastrophizing. Thus, perceived stress may be relevant to the PFP experience, but the present findings do not support an association with knee function after adjustment for the other included covariates.

BMI showed a similar pattern. Higher BMI was associated with poorer knee function in the unadjusted analysis, but not after adjustment. Hart et al. [27] found that adults with PFP had a slightly higher BMI than pain-free controls. However, prospective evidence did not establish BMI as a significant risk factor for developing PFP, and BMI was not associated with intervention outcomes [27]. Collins et al. [5] likewise found that BMI was generally not associated with subsequent pain or functional outcomes in people with PFP. The results suggest that BMI may vary for adults with and without PFP, but may be relatively limited to explain function in those with existing symptoms. This may be particularly important in the gym population, as in this study, as BMI does not differentiate between adipose tissue and lean mass [37].

History of knee pain (first-time vs. recurrent pain) was not associated with knee function in either the unadjusted or adjusted analysis. This is not directly comparable to the literature available, which has focused on the duration of symptoms, not recurrence. Collins et al. [3] found that longer symptom duration was the most consistent predictor of poor recovery at 12 months, with symptoms lasting longer than 2 months being linked to unfavorable outcome. At 5–8 year follow-up, Lankhorst et al. [4] reported that a higher level of symptoms after 12 months was associated with greater pain, while a lower baseline AKPS score was associated with poorer long-term function. A binary recurrence measure does not account for the length of the current episode, number of prior episodes, symptom-free periods, or the total symptom burden. The absence of an association with knee function may therefore partly reflect the limited sensitivity of this measure rather than the absence of a clinically relevant relationship. Future studies should characterize symptom history in greater detail.

### 4.4. Clinical Implications

The primary finding of this study was that pain catastrophizing remained associated with knee function after adjustment for perceived stress, BMI, and knee pain history. This finding is consistent with existing recommendations to consider psychological factors when assessing individuals with PFP, but the present study cannot determine whether screening itself improves clinical outcomes. Pain catastrophizing, pain self-efficacy and fear-avoidance beliefs were the only psychological factors that were found to be clinically relevant for examination, treatment planning and prognosis, and were also identified as research priorities [8]. This is also consistent with current guidelines recommending screening for psychological factors such as catastrophizing as it can be associated with prognosis [14]. The findings from this study add evidence from predominantly male, physically active individuals and suggest that catastrophizing may be relevant across different PFP populations.

The present study cannot establish whether catastrophizing is a modifiable treatment target. However, previous intervention studies have examined psychologically informed approaches in people with PFP. A mindfulness program combined with exercise therapy resulted in significantly lower levels of catastrophizing and higher levels of knee function when compared with exercise therapy alone in female runners with PFP [38]. Similarly, a group of adolescents with PFP who received exercise-based physiotherapy with the support of educational videos showed greater improvement in function, pain, and maladaptive beliefs [39]. These findings indicate that psychologically informed interventions may have the potential to lower catastrophizing in individuals with PFP. However, the evidence presented is largely related to female runners as well as adolescents, and its applicability to the physically active adults in the present study remains unclear.

More broadly, these findings are consistent with the increasing recognition that PFP should be considered beyond a narrowly biomechanical framework, with psychological factors considered alongside physical and clinical features [6,7,8,14]. The observed association between pain catastrophizing and knee function further supports considering psychological factors within personalized rehabilitation, consistent with recent best-practice recommendations emphasizing treatment selection based on the individual patient’s symptoms, needs, and preferences [8,14,40]. When elevated catastrophizing or other relevant psychological factors are identified, current clinical guidance supports approaches such as tailored education, reassurance, graded exposure, cognitive–behavioral strategies, or referral to an appropriate health professional when indicated [14]. Previous intervention studies also suggest that psychologically informed approaches can be integrated with exercise-based rehabilitation, although the available evidence has primarily involved female runners and adolescents [38,39]. Given that longer symptom duration is consistently associated with poorer prognosis in PFP [3,4], comprehensive assessment early in the course of care may be particularly relevant to a recovery-oriented approach to care. However, the present cross-sectional findings do not establish catastrophizing as a prognostic marker for persistent disability or demonstrate that targeting catastrophizing improves long-term outcomes.

### 4.5. Strengths and Limitations

This study has several strengths. It explored the relationship between pain catastrophizing and knee function among a predominantly male, physically active sample of individuals who are underrepresented in previous studies of PFP. Instead of using univariable associations, this study investigated the association of catastrophizing with knee function in the same multivariable model, which included other factors, such as perceived stress, BMI, and history of knee pain. The low variance inflation factors indicated that multicollinearity was unlikely to have materially affected the estimates.

Several limitations should be considered. The cross-sectional design prevents conclusions about causality or the direction of the associations, and reverse causation is possible; poorer knee function may contribute to greater catastrophizing. Longitudinal studies are therefore needed to clarify the temporal relationship between pain catastrophizing and knee function. Recruitment through convenience sampling and online survey distribution may also have introduced selection bias and limited the representativeness of the sample. BMI was calculated from self-reported height and weight, which may have introduced measurement error. No formal a priori sample-size calculation was performed, and the modest sample size limited the precision of the regression estimates and the number of predictors that could be examined. The study may therefore have been insufficiently powered to detect smaller associations, and the regression estimates should be interpreted as exploratory. Larger studies would allow more precise estimates and examination of a broader range of potentially relevant factors. Eligibility was based on self-reported symptoms consistent with PFP rather than clinical examination, which may have resulted in misclassification and greater sample heterogeneity. Although screening followed the consensus definition [14] and age was restricted to 18–40 years, future studies should clinically confirm PFP and exclude alternative diagnoses where feasible. Not all potentially relevant psychological factors were assessed, including kinesiophobia and pain self-efficacy [8]. Residual confounding by these and other unmeasured psychological constructs is therefore possible, and the observed association with catastrophizing should not be interpreted as independent of the broader psychological profile. Future studies should examine these constructs alongside catastrophizing to better characterize the broader psychological profile associated with knee function. Finally, the predominantly male, physically active sample, with relatively low catastrophizing and moderate-to-good knee function, may limit generalizability to women, less active individuals, and those with more severe symptoms or functional limitations. The relatively high functional status and restricted range of self-reported scores may also have reduced variability and limited the ability to detect associations with knee function. The magnitude of this effect cannot be determined from the present data. Replication in more diverse PFP populations is warranted.

## 5. Conclusions

In this cross-sectional study of predominantly male, physically active adults with self-reported symptoms of PFP, greater pain catastrophizing remained associated with poorer knee function after adjustment for perceived stress, BMI, and knee pain history. Perceived stress and BMI were associated with knee function only in the univariable analyses, whereas knee pain history was not associated with function. These findings support considering pain catastrophizing within a broader biopsychosocial assessment of PFP. Prospective studies are needed to clarify the direction of this association and determine whether psychologically informed interventions provide additional benefit in this population.

## Figures and Tables

**Table 1:** Characteristics of the study participants (n = 55).

Variable	Mean (SD)	Median (IQR)	*n* (%)
Age, years	27.38 (7.16)	25.00 (21.50, 35.00)	
Gender			
Male			49 (89%)
Female			6 (11%)
BMI, kg/m^2^	26.06 (5.17)	25.95 (22.64, 29.40)	
PCS (0–52)	12.25 (10.67)	10.00 (5.00, 16.00)	
PSS (0–40)	16.13 (5.66)	17.00 (12.50, 20.00)	
AKPS (0–100)	78.24 (10.41)	77.00 (72.00, 85.50)	

BMI: body mass index; PCS: Pain Catastrophizing Scale; PSS: Perceived Stress Scale; AKPS: Anterior Knee Pain Scale. Higher AKPS scores indicate better knee function and lower pain severity; *n*: number of participants.

**Table 2:** Unadjusted associations between candidate predictors and anterior knee pain scale (AKPS) outcome (n = 55).

Predictor	β	SE	T Value	df	*p* Value	Low 95% CI	Upper 95% CI	R^2^
PCS (0–52)	−0.48	0.12	−4.13	53	<0.001	−0.72	−0.25	0.23
PSS (0–40)	−0.70	0.23	−3.00	53	0.004	−1.17	−0.23	0.13
BMI	−0.58	0.26	−2.21	53	0.032	−1.11	−0.05	0.07
Recurrent knee pain	−4.02	3.29	−1.22	53	0.227	−10.62	2.57	0.01

PCS: Pain Catastrophizing Scale; PSS: Perceived Stress Scale; BMI: body mass index; CI: confidence interval.

**Table 3:** Adjusted associations between candidate predictors and anterior knee pain scale (AKPS) outcome (*n* = 55).

Predictor	β	SE	T Value	df	*p* Value	Low 95% CI	Upper 95% CI	Adjusted R^2^
PCS (0–52)	−0.36	0.12	−2.90	50	0.005	−0.60	−0.11	0.28
PSS (0–40)	−0.43	0.23	−1.88	50	0.065	−0.90	0.03	
BMI	−0.35	0.24	−1.46	50	0.152	−0.84	0.13	
Recurrent knee pain	−2.31	2.92	−0.79	50	0.433	−8.17	3.55	

PCS: Pain Catastrophizing Scale; PSS: Perceived Stress Scale; BMI: body mass index; CI: confidence interval.

## Data Availability

The data cannot be made publicly available because the participant consent and ethical approval did not explicitly cover unrestricted public sharing of individual-level research data. To protect participant confidentiality, deidentified data may instead be made available from the corresponding author upon reasonable request and subject to applicable ethical requirements.

## Supplementary Materials

The following supporting information can be downloaded at https://www.mdpi.com/article/10.3390/healthcare14193212/s1, The STROBE checklist for cross-sectional studies is provided as Supplementary File S1.

## Abbreviations

The following abbreviations are used in this manuscript:

AKPS	Anterior Knee Pain Scale
PFP	Patellofemoral pain
BMI	Body mass index
PCS	Pain Catastrophizing Scale
PSS	Perceived Stress Scale
